# Multidisciplinary Trends in Endoscopic Surgery in Japan: Insights From the 17th Nationwide Survey

**DOI:** 10.1111/ases.70302

**Published:** 2026-04-26

**Authors:** Hidefumi Shiroshita, Shuji Takiguchi, Fuminori Taniguchi, Nobuyuki Hinata, Tetsuji Uemura, Susumu Eguchi, Yukinori Kurokawa, Yusuke Kinugasa, Tomonori Akagi, Shunsuke Tsukamoto, Aya Kanehira, Yosuke Seki, Satoshi Ieiri, Masayuki Iwazaki, Yukio Sato, Kaoru Kitamura, Akihiro Nakajo, Kazuma Okamoto, Akira Miyajima, Hiroshi Takahashi, Mitsuhisa Takatsuki, Toshikazu Takeda, Katsunori Fukutake, Yasushi Hoshikawa, Isao Matsumoto, Shigeo Akira, Masafumi Inomata, Seigo Kitano, Masahiko Watanabe, Yoshiharu Sakai, Yuko Kitagawa

**Affiliations:** ^1^ Academic Committee of Japan Society for Endoscopic Surgery Tokyo Japan; ^2^ Department of Advanced Medical Personnel Nurturing Oita University Faculty of Medicine Yufu Japan; ^3^ Department of Gastroenterological Surgery Nagoya City University Graduate School of Medical Science Nagoya Japan; ^4^ Department of Obstetrics and Gynecology Tottori University Hospital Yonago Japan; ^5^ Department of Urology Hiroshima University Graduate School of Biomedical and Health Sciences Hiroshima Japan; ^6^ Department of Plastic and Reconstructive Surgery, Faculty of Medicine Saga University Saga Japan; ^7^ Department of Surgery Nagasaki University Graduate School of Biomedical Sciences Nagasaki Japan; ^8^ Department of Gastroenterological Surgery Osaka University Graduate School of Medicine Osaka Japan; ^9^ Department of Gastrointestinal Surgery Institute of Science Tokyo Tokyo Japan; ^10^ Department of Gastroenterological and Pediatric Surgery Oita University Faculty of Medicine Yufu Japan; ^11^ Department of Colorectal Surgery National Cancer Center Hospital Tokyo Japan; ^12^ Department of Surgery Medical Topia Soka Hospital Soka Japan; ^13^ Weight Loss and Metabolic Surgery Center, Yotsuya Medical Cube Tokyo Japan; ^14^ Department of Pediatric Surgery, Research Field in Medical and Health Sciences, Medical and Dental Area, Research and Education Assembly Kagoshima University Kagoshima Japan; ^15^ Department of General Thoracic Surgery Tokai University School of Medicine Isehara Japan; ^16^ Department of Thoracic Surgery University of Tsukuba Ibaraki Japan; ^17^ AM Clinic Fukuoka Japan; ^18^ Department of Digestive Surgery, Breast and Thyroid Surgery Kagoshima University Kagoshima Japan; ^19^ Department of Cardiovascular Surgery Hamamatsu University School of Medicine Hamamatsu Japan; ^20^ Komazawa Urology Tokyo Japan; ^21^ Department of Orthopedic Surgery Toho University School of Medicine Tokyo Japan; ^22^ Department of Digestive and General Surgery, Graduate School of Medicine University of the Ryukyus Ginowan Japan; ^23^ Department of Urology Keio University School of Medicine Tokyo Japan; ^24^ Department of Thoracic Surgery Fujita Health University Toyoake Japan; ^25^ Department of Thoracic Surgery Kanazawa University Kanazawa Japan; ^26^ Meirikai Tokyo Yamato Hospital Tokyo Japan; ^27^ Oita University Yufu Japan; ^28^ Nishiazabu Medical Clinic Tokyo Japan; ^29^ Japanese Red Cross Osaka Hospital Osaka Japan; ^30^ Department of Surgery Keio University School of Medicine Tokyo Japan

**Keywords:** endoscopic surgery, Japan society for endoscopic surgery, national survey

## Abstract

The Japan Society for Endoscopic Surgery (JSES) conducts a nationwide survey every 2 years to evaluate the status and trends of endoscopic surgery in Japan. This article reports the results of the 17th Nationwide Survey conducted by the JSES in 2022 and 2023. A questionnaire assessing the current status of endoscopic surgery was distributed to institutions nationwide. Since 1990, a total of 4 286 446 endoscopic procedures have been performed in Japan, with a continuous increase in most surgical fields. Although the number of procedures temporarily declined during the COVID‐19 pandemic, endoscopic surgical activity steadily recovered and increased across all disciplines. Furthermore, the use of robot‐assisted surgery has expanded rapidly in multiple specialties, reflecting broader clinical adoption and technological progress. These nationwide data demonstrate that endoscopic surgery is widely and safely performed across all surgical specialties in Japan. The findings of this multidisciplinary survey provide valuable insights into current practice patterns and will contribute to further development, standardization, and safe dissemination of endoscopic surgery.

## 1 Introduction

Endoscopic surgery with laparoscopic cholecystectomy was introduced in Japan in 1990 and has since become widely adopted because of its minimally invasive nature. With advances in surgical techniques and devices, the indications and target organs for endoscopic surgery have expanded across multiple surgical specialties, and laparoscopic cholecystectomy is now established as the standard treatment for cholecystolithiasis [1, 2]. More recently, the introduction and dissemination of robot‐assisted surgery, supported by expanded insurance coverage, have further influenced endoscopic surgical practice across various clinical fields in Japan.

Since 1990, the Japan Society for Endoscopic Surgery (JSES) has conducted a nationwide survey every 2 years to evaluate the status and trends of endoscopic surgery in Japan [3, 4, 5, 6, 7, 8]. In this article, we present the results of the 17th JSES Nationwide Survey conducted between 2022 and 2023, focusing on multidisciplinary trends across surgical specialties.

## 2 Questionnaire Survey Conducted by the JSES

A questionnaire on the current status of endoscopic surgery in 10 surgical domains was sent to 3759 institutions in Japan, including 3223 JSES member facilities and 536 core educational facilities from the Japanese Urological Association. The questionnaire included questions on the number of patients undergoing endoscopic procedures, the indications for each procedure, and the incidence of intraoperative and postoperative complications. Overall, 1330 of the 3759 institutions surveyed responded, with a response rate of 35.4%. In the field of gynecology, survey results from the Japan Society of Gynecologic and Obstetric Endoscopy and the Minimally Invasive Therapy (JSGOE) Survey for 2022 and 2023 are shown, together with the results of the current survey. In addition, a questionnaire survey was conducted using the JSES questionnaire, led by the Japan Society of Plastic Surgery; the results of the 2022 and 2023 surveys of the Japan Society of Plastic Surgery were added to the survey results. The data were analyzed for all domains, including abdominal, thoracic, mammary, and thyroid glands; cardiovascular; obstetrics and gynecology; and urologic, orthopedic, and plastic surgery.

## 3 Number of Patients Undergoing Endoscopic Surgery

**FIGURE 1:**
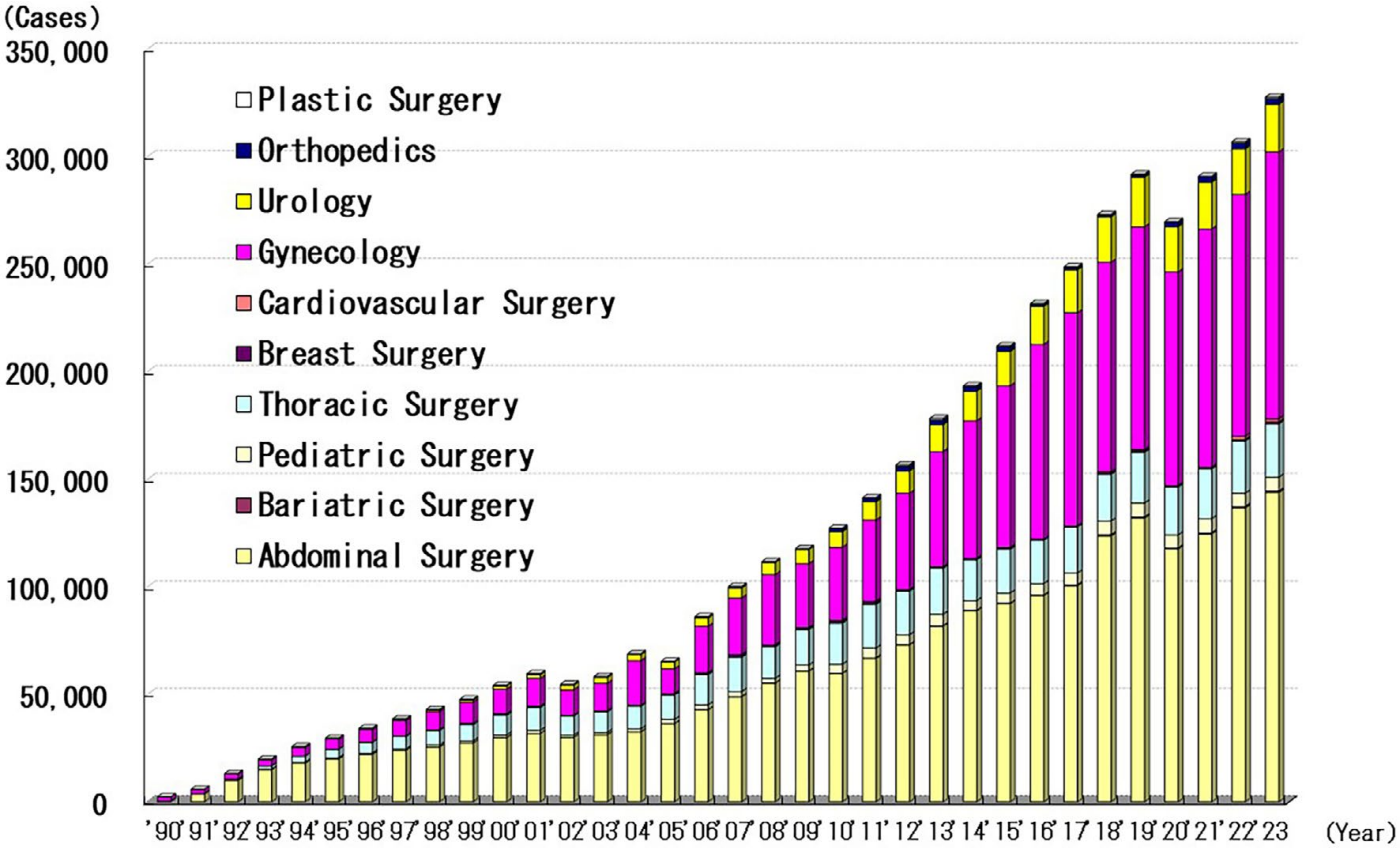
Annual number of endoscopic surgeries.

The number of patients undergoing endoscopic surgery increased across most surgical domains (Figure 1). Between 1990 and 2023, 4 286 446 patients underwent endoscopic surgery in Japan. Of these, 2 002 776 underwent endoscopic abdominal surgery, 1 386 899 underwent endoscopic obstetric and gynecologic surgery, 457 345 underwent endoscopic thoracic surgery, and 275 002 underwent endoscopic urological surgery.

## 4 Robotic Surgery

**FIGURE 2:**
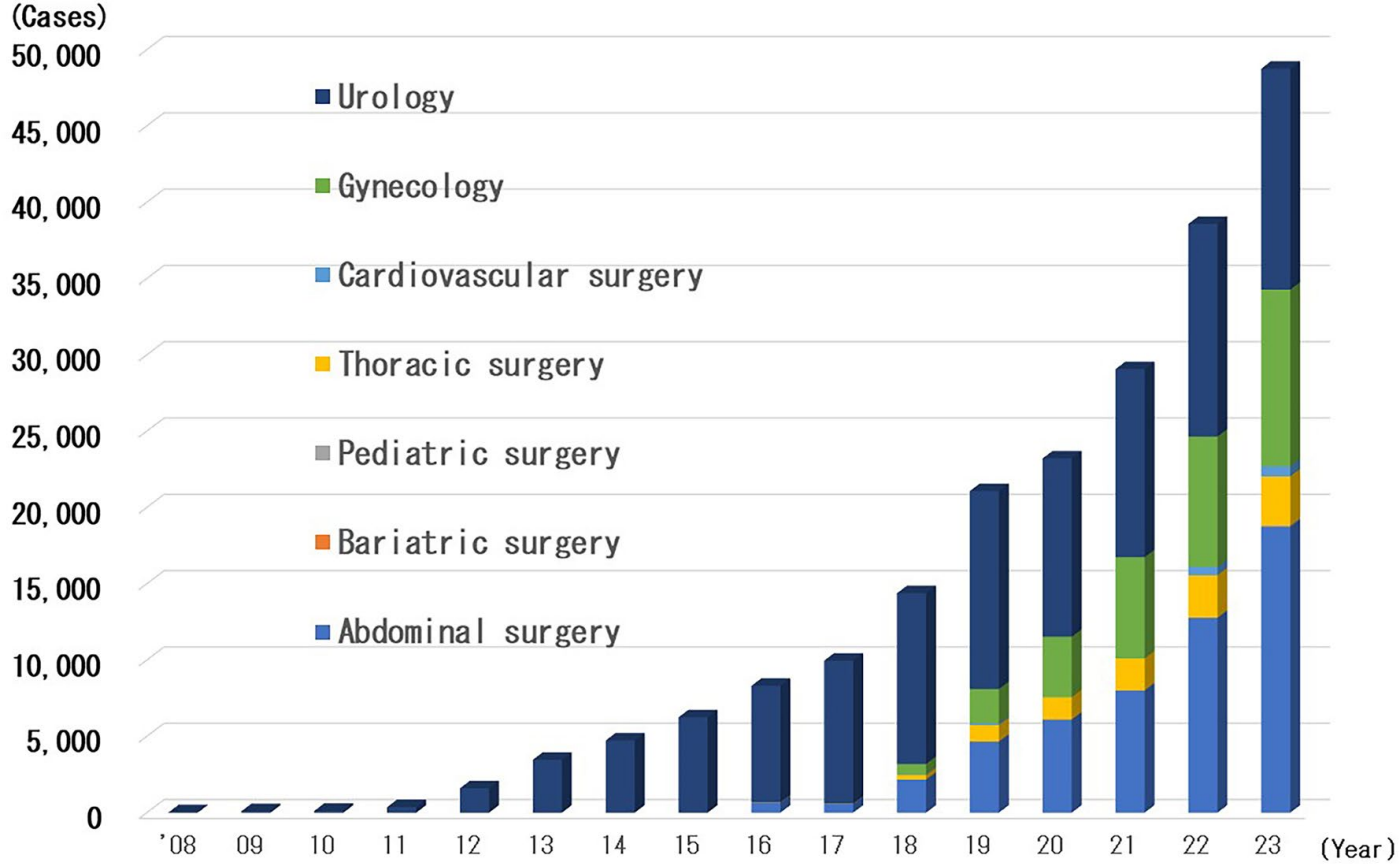
Annual number of robotic surgeries.

The number of robotic surgeries has increased rapidly since 2018. By the end of 2023, 209 993 patients had undergone robotic surgery (Figure 2). In 2023, 48 753 patients underwent robotic surgery. Of these, 17 740 underwent robotic abdominal surgery, 14 478 underwent robotic urological surgery, 11 575 underwent robotic gynecological surgery, 3226 underwent robotic thoracic surgery, 665 underwent robotic cardiovascular surgery, 65 underwent robotic pediatric surgery, and 4 underwent robotic bariatric surgery.

## 5 Abdominal Surgery

### 5.1 Trends in the Number of Laparoscopic Abdominal Surgeries Performed Over Time

**FIGURE 3:**
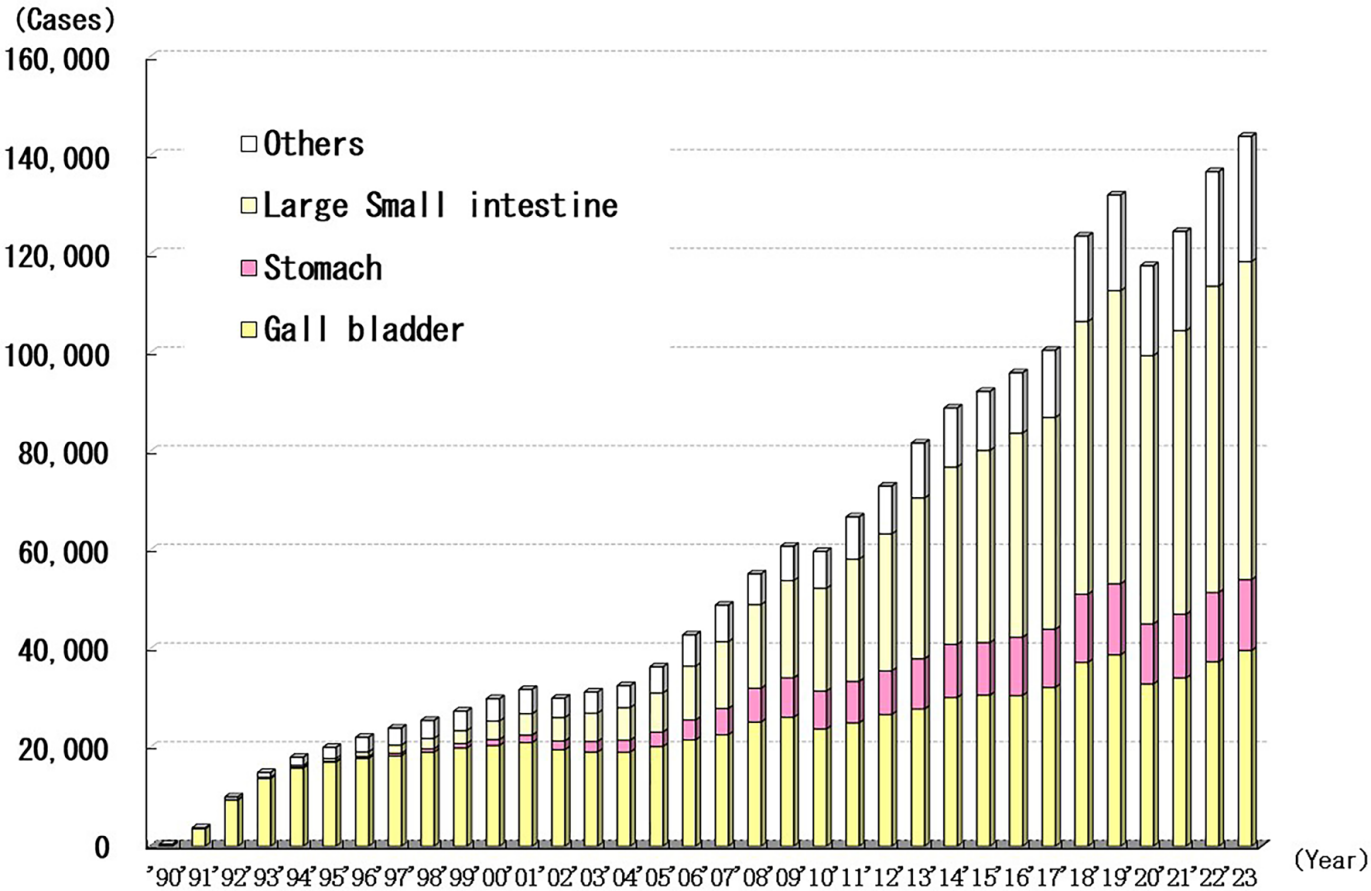
Annual number of laparoscopic procedures for abdominal disease.

The number of laparoscopic abdominal surgeries has increased annually since 1990. By the end of 2023, 2 002 776 patients had undergone laparoscopic abdominal surgery (Figure 3). Although only 381 laparoscopic abdominal procedures were performed in 1990, 143 867 were performed by 2023. A total of 796 517 patients underwent laparoscopic cholecystectomy, which accounted for 39.8% of the total number of abdominal surgeries performed. The next most performed procedures were laparoscopic resection of the small intestine, colon, and rectum (721 027 cases, 36.0%) and laparoscopic resection of the stomach (200 978 cases, 10.0%).

### 5.2 Selection of Pneumoperitoneum or Abdominal Wall‐Lifting Methods

Among 521 institutions surveyed, 506 (97.1%) routinely employed the pneumoperitoneum method, while two institutions (0.4%) employed the pneumoperitoneum and abdominal wall‐lifting methods on a case‐by‐case basis. Recently, the abdominal wall‐lifting method has been used less frequently than the pneumoperitoneum method; however, it remains an important option for patients with deteriorated cardiopulmonary function.

### 5.3 Single‐Port Laparoscopic Surgery

Single‐port laparoscopic surgery was first performed in Japan in 2008, and by the end of 2023, 124 643 patients had undergone this procedure. Single‐port surgeries accounted for 6.0% (8601/143867) of all laparoscopic abdominal surgeries in 2023. The most performed single‐port laparoscopic surgery was cholecystectomy, which was performed on 3010 patients in 2023. The next most performed procedures were appendectomy, inguinal herniorrhaphy, and colon and rectal resection.

### 5.4 Port‐Site Recurrence

From 1990 to 2021, port site recurrence occurred in 150 patients in Japan (colon cancer in 50, gallbladder cancer in 43, gastric cancer in 19, rectal cancer in 18, hepatocellular carcinoma in four, gastric gastrointestinal stromal tumor in three, jejunal cancer in two, pancreatic cancer in three, duodenal cancer in two, cholangiocellular carcinoma in two, bile duct cancer in one, esophageal cancer in one, metastatic tumor of the abdominal wall in one, and unknown primary cancer in one). In 2022 and 2023, port site recurrence occurred in 27 patients (colon cancer in 18, gastric cancer in two, rectal cancer in three, gallbladder cancer in two, and pancreatic cancer in two).

## 6 Current Status of Endoscopic Surgery in Each Domain

### 6.1 Laparoscopic Cholecystectomy

**FIGURE 4:**
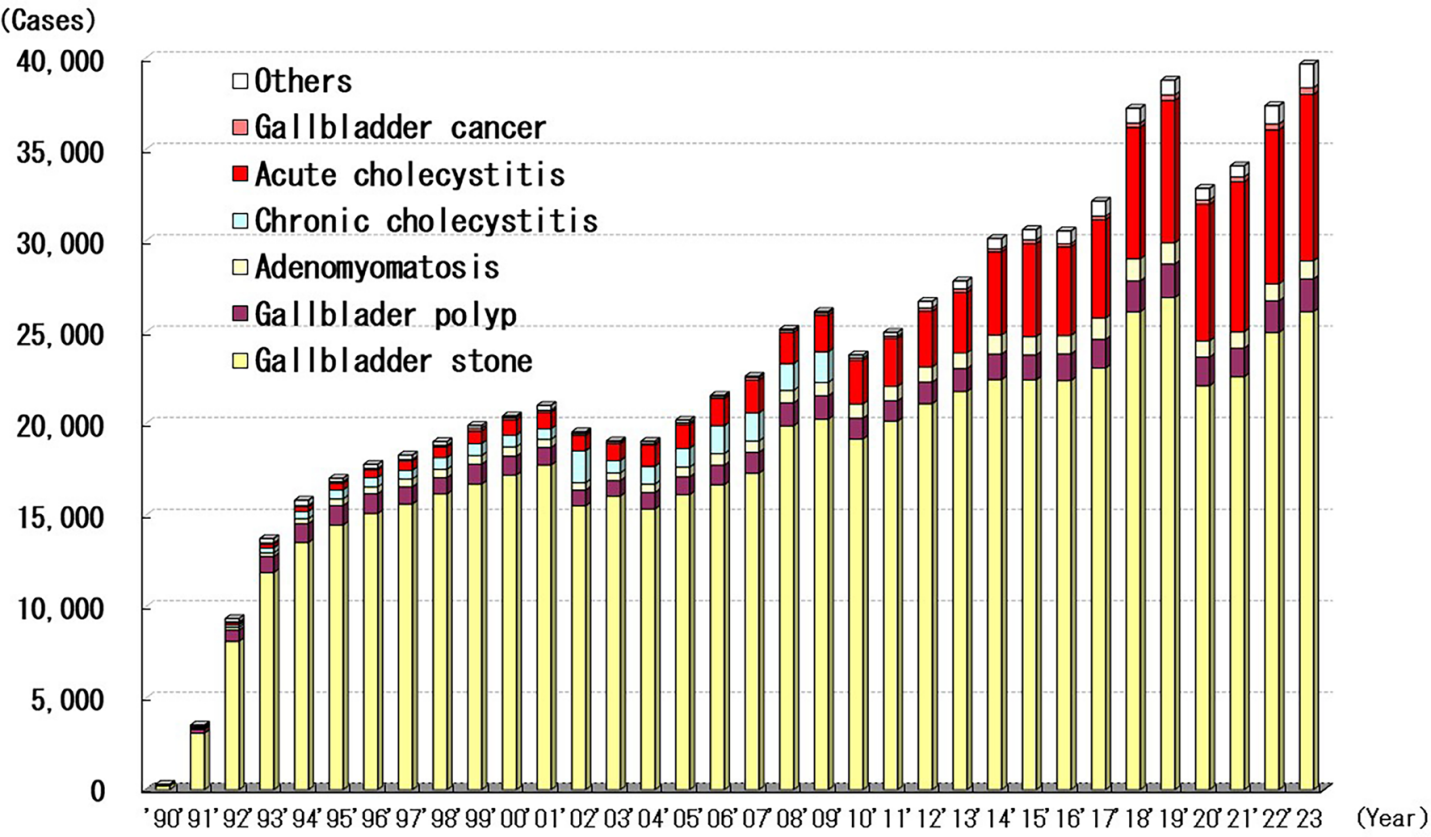
Annual number of laparoscopic cholecystectomy.

Laparoscopic cholecystectomy was first reported in Japan in 1990. Between then and 2023, 796 517 patients underwent this procedure. The number of patients who underwent this procedure annually and the diseases in which this procedure was performed are shown in Figure 4. In 1990, only 299 patients underwent laparoscopic cholecystectomy; however, In 2023, this type of surgery had been performed in 39 681 patients. In 2023, open cholecystectomies were performed in 2422 patients, and 94.2% (39 681/42103) of all cholecystectomies were performed laparoscopically. Of the 796 517 patients who underwent laparoscopic cholecystectomy, 3258 (0.4%) required intraoperative hemostasis via laparotomy, and 604 (0.08%) required postoperative hemostasis. The most common sites of bleeding were the liver bed and the cystic artery, which together accounted for > 80% of cases of bleeding. In total, 4206 patients (0.5%) suffered from bile duct injury, and 286 (0.04%) had postoperative bile duct stenosis. Conversion to open surgery was required in 23 199 patients (2.9%) for reasons not associated with bleeding, bile duct injury, or other organ injuries. The most common reason was indistinct and severe adhesions due to local inflammation, which necessitated conversion to open surgery in a combined total of 17 377 patients.

### 6.2 Laparoscopic Surgery for Bile Duct Stones

A total of 60 593 patients underwent laparoscopic treatment for bile duct stones by the end of 2023, 2277 of whom were treated in 2023. Laparoscopic common bile duct exploration was performed in 13 009 of the 60 593 patients (21.5%), and laparoscopic transcystic choledocholithotomy was performed in 2144 (3.5%). Laparoscopic cholecystectomy combined with either pre‐ or postoperative endoscopic sphincterotomy or endoscopic papillary balloon dilatation was performed in 44 747 patients (73.8%). Recently, the use of preoperative endoscopic sphincterotomy followed by laparoscopic cholecystectomy has gained popularity; this combination method accounted for 72.1% of all laparoscopic surgeries for bile duct stones in 2023.

### 6.3 Laparoscopic Inguinal Hernia Repair

In total, 589 704 inguinal hernia repairs were performed between 1990 and 2023. Among the 492 institutions, 248 performed endoscopic procedures on all patients, 93 on patients with bilateral hernia, and 72 on patients with recurrent hernia. Endoscopic procedures were performed on 221 011 patients (37.5%): 170 658 (28.9%) underwent transabdominal preperitoneal (TAPP) repair, 49 762 (8.4%) underwent total extraperitoneal (TEP) repair, and 591 (0.1%) underwent robotic surgery. Endoscopic procedure‐related complications occurred in 12 118 (5.5%) patients, including 721 (0.3%) who required intraoperative conversion to an open procedure or who underwent postoperative laparotomy. The recurrence rates in patients who underwent TAPP repair and TEP repair between 2022 and 2023 were 0.2% (63/26595 patients) and 0.2% (17/8271 patients), respectively, across 143 institutions.

### 6.4 Laparoscopic Abdominal Wall Hernia Repair

In total, 7687 patients underwent laparoscopic repair of abdominal wall hernias between 2020 and 2023. The most common indication for laparoscopic repair was incisional hernia (5837 cases), followed by umbilical hernia. From 2020 to 2023, 46.7% (7687/16444) of all abdominal wall hernia repairs were performed laparoscopically. Laparoscopic parastomal hernia repair was performed in 256 patients between 2020 and 2023. Postoperative complications (seroma and chronic pain) were reported in 255 patients. Conversion from laparoscopic to open surgery was required in 0.4% (27/7687) of patients.

### 6.5 Laparoscopic Surgery for Esophageal Disease

A total of 52 299 laparoscopic esophageal procedures were performed between 1990 and 2023, with 4833 procedures in 2023. From 2018 to 2023, the number of robot‐assisted esophagectomy procedures for esophageal cancer increased annually, reaching 1296 in 2023.

### 6.6 Gastric and Duodenal Ulcers

A total of 17 848 patients underwent laparoscopic surgery for gastric or duodenal ulcers. The most performed procedure was closure of duodenal ulcer perforation (11 620 patients, 65.1%). In 2023, 973 endoscopic procedures and 887 open procedures were performed; therefore, 52.3% of operations for gastric and duodenal ulcer perforations were performed endoscopically. The proportion of endoscopic surgeries performed for gastric and duodenal ulcer perforations did not increase significantly over time.

### 6.7 Gastric Submucosal Tumors

In total, 22 285 patients underwent laparoscopic surgery for gastric submucosal tumors. Of these patients, 14 319 (64.3%) were diagnosed with a gastrointestinal stromal tumor. The proportion of endoscopic surgeries in which the wedge resection method (1019/1681 procedures in 2023) is employed has increased annually. Moreover, the proportion of gastric submucosal tumor surgeries performed endoscopically substantially increased in this field, and 88.4% of operations (1681/1901) were endoscopic in 2023. Furthermore, laparoscopic endoscopic cooperative surgery was performed on 491 patients in 2023.

### 6.8 Gastric Cancer

**FIGURE 5:**
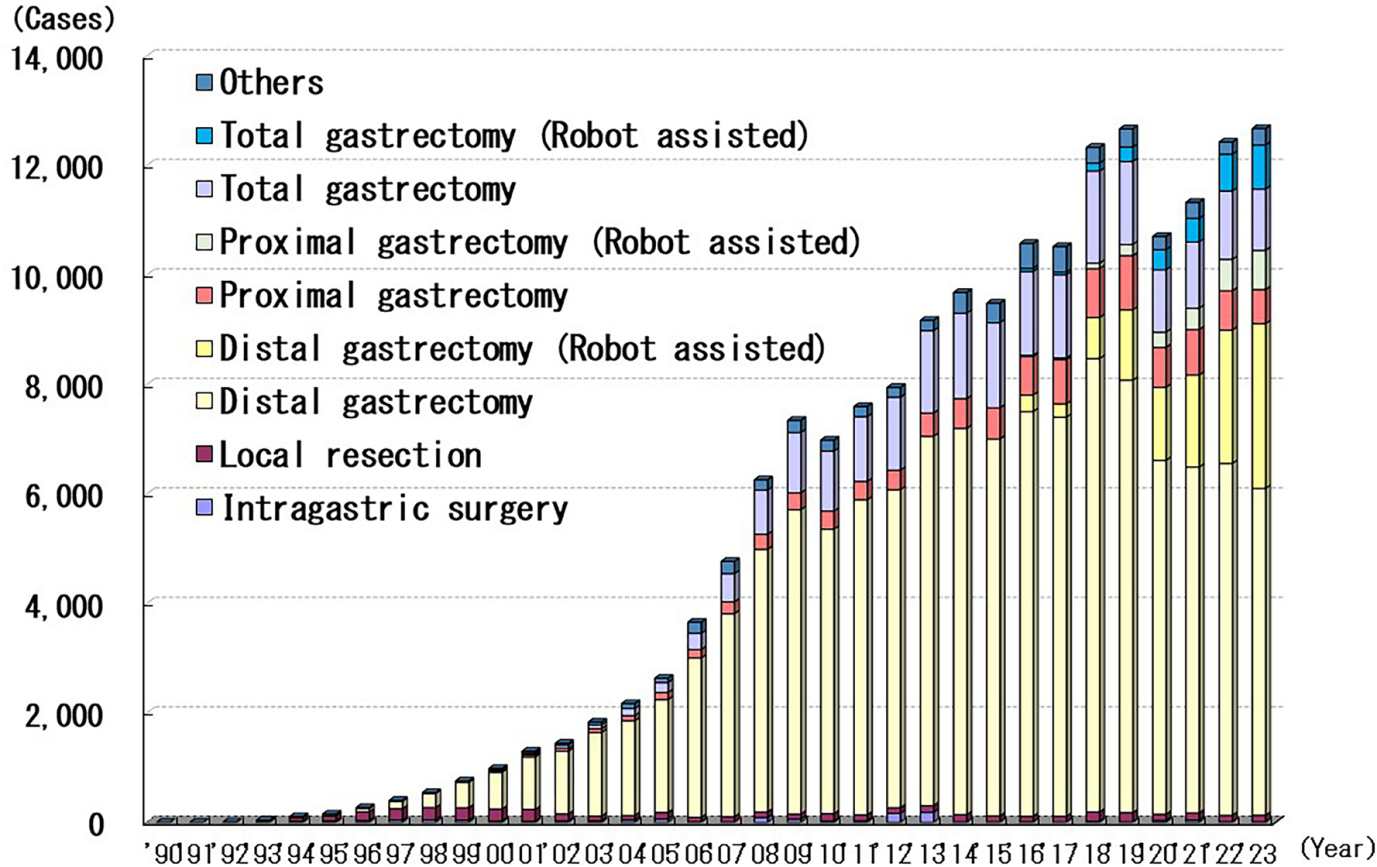
Annual number of laparoscopic procedures for gastric cancer.

A total of 178 693 laparoscopic gastrectomies were performed between 1991 and 2023; 12 673 of these were performed in 2023 (Figure 5). Currently, the total number of laparoscopic gastrectomies has increased as this procedure is less invasive than open gastrectomy. The number of laparoscopic gastrectomies declined in 2020 and will recover slightly in 2021. Furthermore, the number of robot‐assisted gastrectomies has been increasing rapidly each year.

The incidence of intraoperative or postoperative complications in laparoscopic distal gastrectomy was 12.9% in 2023. In contrast, the incidence of these complications in laparoscopy‐assisted total gastrectomy was 19.2% in 2023.

### 6.9 Colorectal Surgery for Benign Disease

Endoscopic surgery for colorectal diseases was initiated in 1991. A total of 721 027 cases of colorectal disease were treated surgically by the end of 2023; 248 969 were benign. The most performed procedure was laparoscopic appendectomy (194 888 patients, 78.3%). Other conditions included complicated diverticulitis (12 317 cases, 4.9%), benign tumors (11 269 cases, 4.5%), rectal prolapse (6777 cases, 2.7%), ulcerative colitis (5353 cases, 2.2%), and Crohn's disease (5231 cases, 2.1%). The number of laparoscopic surgeries performed for benign colorectal diseases has increased annually.

### 6.10 Colorectal Surgery for Malignant Disease

**FIGURE 6:**
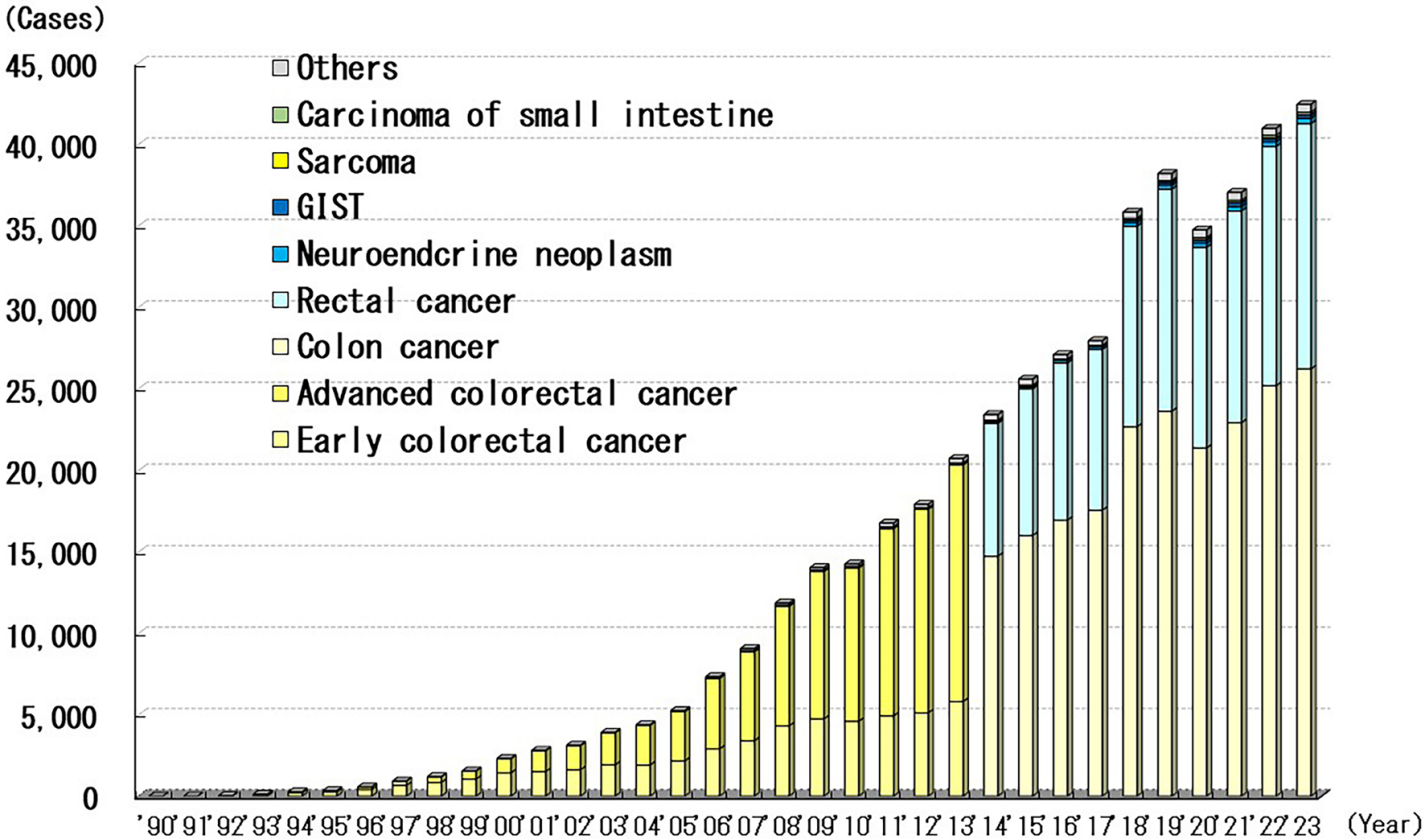
Annual number of laparoscopic procedures for small and large intestine disease (malignancy).

A total of 472 058 patients with malignant colorectal disease were surgically treated by the end of 2023 (Figure 6). The proportion of endoscopic colorectal cancer surgeries increased from 1993 to 2019; however, it decreased in 2020 and recovered slightly by 2022. In 2023, 83.9% (26 211/31248) of all colon cancer surgeries were performed laparoscopically. Furthermore, in 2023, 90.5% (15 045/16628) of all rectal cancer surgeries were performed laparoscopically.

### 6.11 Liver Disease

**FIGURE 7:**
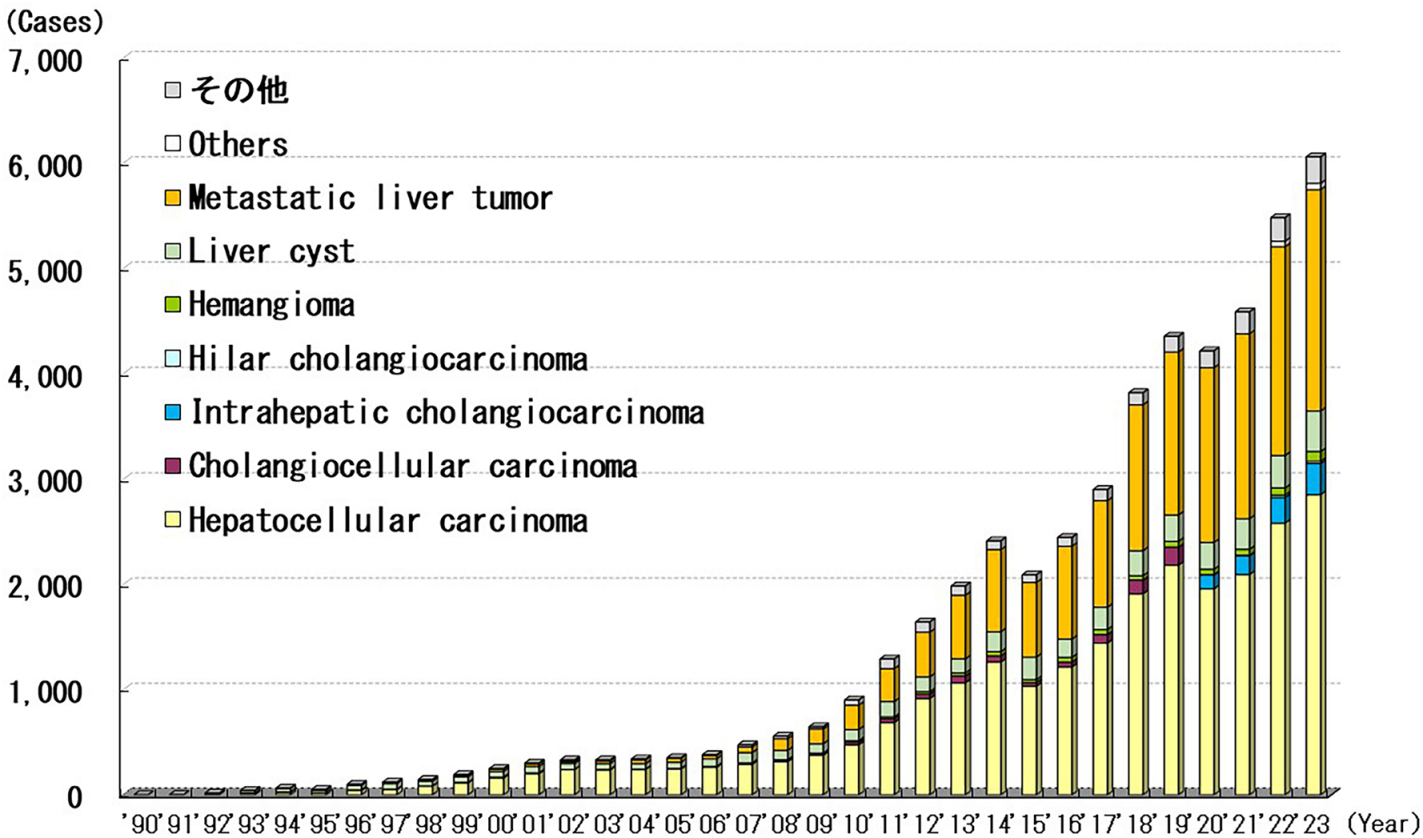
Annual number of laparoscopic procedures for liver disease.

Between 1990 and 2023, 48 896 laparoscopic liver surgeries were performed (Figure 7). The most common indication for laparoscopic liver surgery was hepatocellular carcinoma (24 592 cases, 50.3%), followed by metastatic liver tumors (15 918 cases, 32.6%), and liver cysts (4006 cases, 8.2%). The annual number of laparoscopic liver resections increased between 2021 and 2023. Among 48 896 patients, 737 experienced intraoperative complications (e.g., hemorrhage), 1040 required conversion to open surgery, and postoperative complications (e.g., bile leak and wound infection) were reported in 2916.

### 6.12 Pancreatic Disease

Between 1990 and 2023, 14 907 patients underwent laparoscopic pancreatic surgery. The most common diseases for which pancreatic surgery was performed were pancreatic cancer (4711 patients, 31.6%), intraductal papillary mucinous neoplasm (3240 patients, 21.7%), and neuroendocrine tumors (2474 patients, 16.6%). The most performed operation was distal pancreatectomy (body‐tail, 10 711 cases, 69.8%; tail, 1113 cases, 7.3%). Pancreaticoduodenectomies were performed in 1308 patients (8.5%). Furthermore, robotic surgery was performed in 1147 patients by 2023. Complications (e.g., pancreatic fistula) occurred in 3690 patients, including 469 who required intraoperative conversion to open surgery or underwent postoperative laparotomy. The proportion of pancreatic surgeries, not only distal pancreatectomies but also pancreatoduodenectomies, has increased annually in recent years.

### 6.13 Splenic Disease

A total of 8751 laparoscopic splenic surgeries were performed, including 8559 splenectomies (97.8%). The number of operations plateaued towards the end of the survey period. In 2022 and 2023, the most common disease for which splenic surgery was performed was benign splenic tumors (126 cases, 27.2%), followed by malignant splenic tumors (119 cases, 25.7%). From 1990 to 2023, 699 complications were reported in 8751 patients (8.0%). Conversion from laparoscopic surgery to open surgery was required in 309 patients.

## 7 Obesity and Metabolic Disorders

Between 2002 and 2023, endoscopic and laparoscopic bariatric procedures were performed in 5273 obese patients. The most performed procedure was laparoscopic sleeve gastrectomy (4009 cases, 76.0%), followed by laparoscopic sleeve gastrectomy with a duodenojejunal bypass (Roux‐en‐Y) (359 patients, 6.8%), laparoscopic Roux‐en‐Y gastric bypass (260 patients, 4.9%), endoscopic intragastric balloon placement (250 patients, 4.7%), and endoscopic sleeve gastroplasty (177 patients, 3.4%). The number of bariatric procedures performed annually increased between 2011 and 2023; in 2023, 645 procedures were performed. Furthermore, robot‐assisted surgery was performed in four cases in 2023. Complications occurred in 172 patients, including 62 who required intraoperative conversion to open surgery or underwent postoperative laparotomy.

## 8 Pediatric Surgery

**FIGURE 8:**
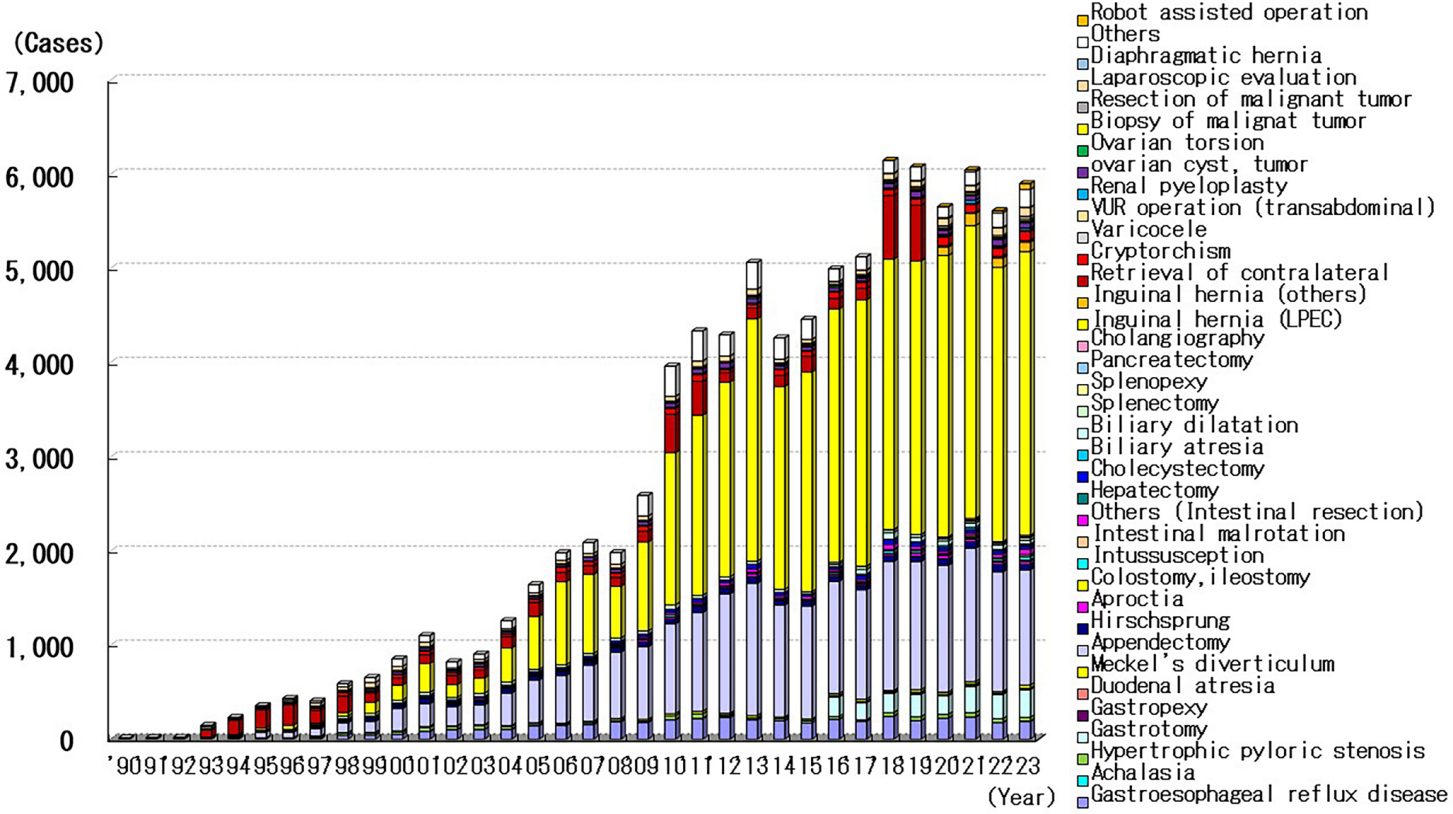
Annual number of endoscopic procedures in pediatric surgery.

The number of laparoscopic surgeries performed in pediatric patients has increased annually, with 89 981 procedures being performed between 1990 and 2023 (Figure 8). The most performed procedure was laparoscopic inguinal hernia repair (41 096 patients, 45.7%), followed by laparoscopic appendectomy (22 116 patients, 24.6%). The number of laparoscopic inguinal hernia repairs performed during this period increased because of the introduction of the laparoscopic percutaneous extraperitoneal closure method (LPEC) for inguinal hernia repair, which was developed in 2005. Thoracoscopic surgery was performed in 7773 patients between 1990 and 2023. In these patients, the most performed procedure was the Nuss procedure to treat pectus excavatum (2294 cases, 29.5%), followed by thoracoscopic surgery for pneumothorax (1766 cases, 22.7%).

## 9 Thoracic Surgery

### 9.1 Thoracoscopic Surgery Methods

Among 215 facilities (multiple responses allowed), the main procedures were as follows: 173 performed complete video‐assisted thoracic surgery (VATS), 38 performed VATS under direct vision, 51 performed a combination of the two or hybrid surgery, and 75 performed robotic surgery. A total of 10 984 robotic surgeries were performed between 2016 and 2023.

### 9.2 Lung Disease

**FIGURE 9:**
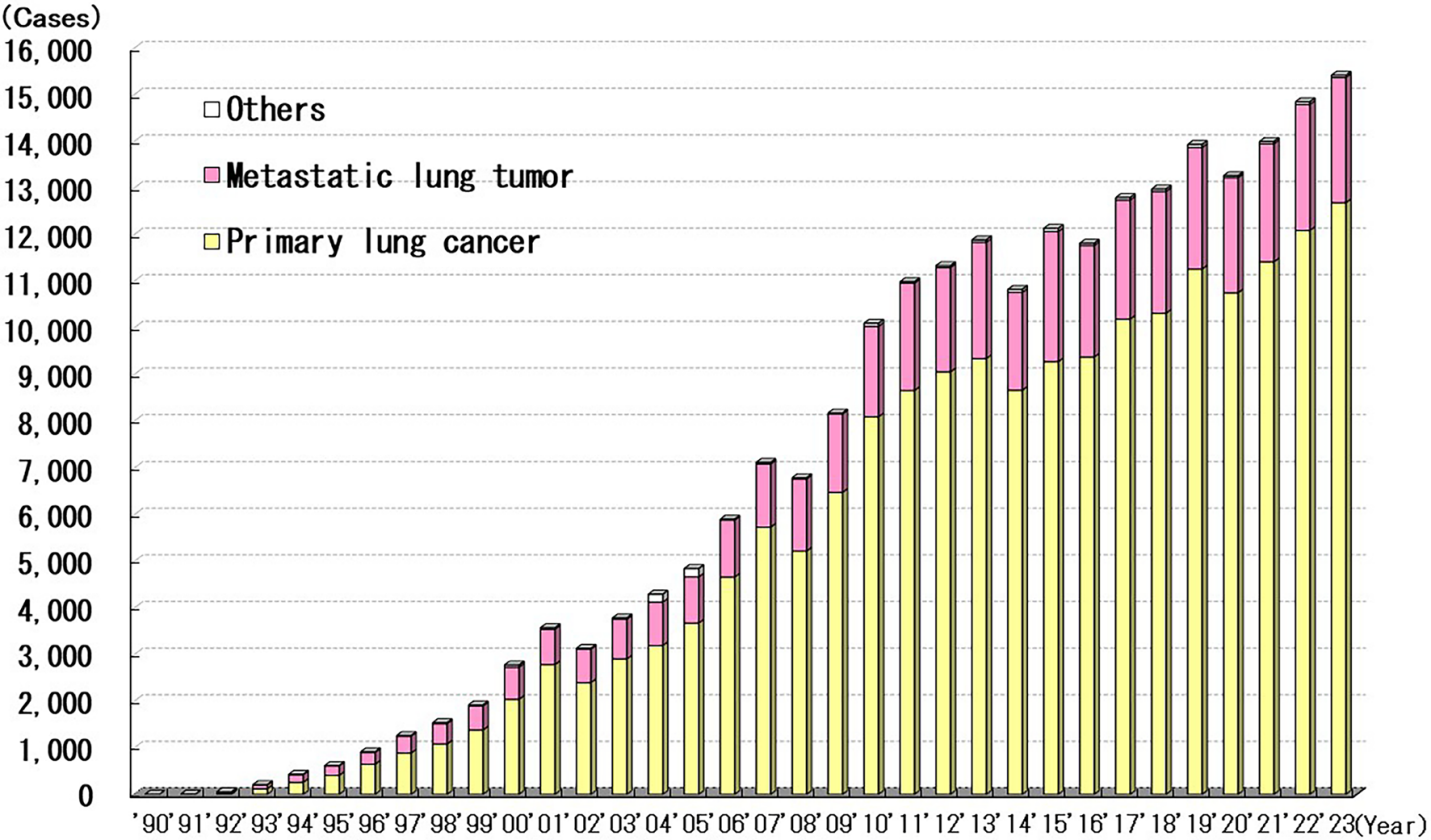
Annual number of laparoscopic procedures for malignant lung disease.

Between 1990 and 2023, 276 396 thoracoscopic surgeries were performed for lung diseases. The number of operations performed for benign and malignant diseases was 42 760 and 233 636, respectively. The number of thoracoscopic surgeries performed for lung disease has increased annually. In particular, the number of surgeries performed annually for lung cancer has increased, with this procedure performed in 12 682 patients in 2023 (Figure 9).

### 9.3 Pleural and Thoracic Wall Disease

A total of 128 223 thoracoscopic surgeries for pleural and thoracic wall diseases were performed between 1990 and 2023. Pneumothorax was the most common indication for these surgeries. In the later years of the survey period, the number of operations performed annually to treat the pyothorax increased.

### 9.4 Mediastinal Disease

A total of 26 026 endoscopic operations for mediastinal disease were performed between 1990 and 2023, including 1539 in 2023. Among 122 facilities, malignant mediastinal tumors and myasthenia gravis were considered contraindications for VATS in 43 and 34 facilities, respectively.

## 10 Mammary Gland and Thyroid Surgery

### 10.1 Mammary Gland Surgery

Between 1995 and 2023, 7842 patients underwent endoscopic surgery for mammary gland diseases. Overall, 1139 and 6703 patients had benign and malignant tumors, respectively. During the survey period, endoscopic surgery for breast cancer was introduced gradually. Breast reconstruction surgery was performed in 846 patients, and breast reconstruction surgery alone in 403 patients.

### 10.2 Thyroid Surgery

A total of 5847 patients underwent endoscopic surgery between 1998 and 2023. This procedure was most performed for benign tumors (2147 cases); however, 1952 patients underwent endoscopic surgery for malignant tumors, and 1348 patients underwent cervical lymphadenectomy for malignant thyroid tumors.

## 11 Cardiovascular Surgery

### 11.1 Cardiovascular Disease

In total, 5468 patients underwent endoscopic cardiovascular surgery by the end of 2023. Of these, 2896 (53.0%) underwent valvuloplasty. Between 1996 and 2023, coronary artery bypass grafting was performed in 275 patients. Furthermore, robot‐assisted surgery was performed in 1368 cases.

### 11.2 Peripheral Vascular Disease

A total of 3299 endoscopic surgeries were performed for peripheral vascular disease. The most performed procedures were endoscopic saphenous vein harvest (1177 cases, 35.7%) and endovascular aneurysm repair (922 cases, 27.9%).

## 12 Obstetrics and Gynecology

**FIGURE 10:**
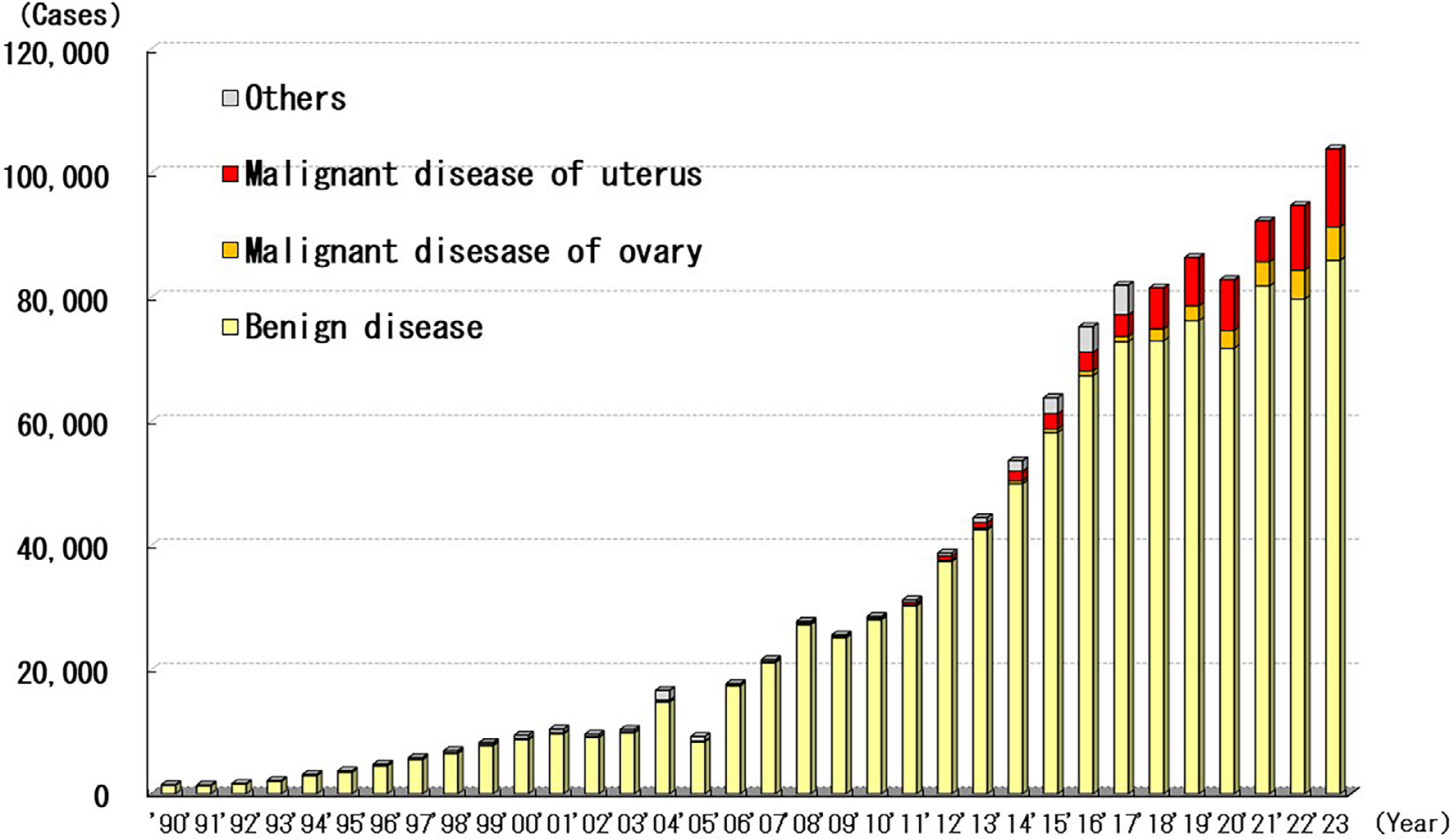
Annual number of laparoscopic procedures for gynecologic disease.

Since 2004, the JSGOE has conducted questionnaire surveys linked to the JSES every other year. In this study, the results of the JSGOE and JSES surveys for 2022 and 2023 were combined (Figure 10).

### 12.1 Laparoscopic Treatment for Uterine and Adnexal Surgery

Between 1990 and 2023, 1 157 578 patients underwent laparoscopic surgery for uterine and adnexal diseases. Of these, 369 281 (31.9%) had uterine myoma. Other diseases included benign ovarian tumors (323 961, 28.0%) and endometriosis (170 600, 14.7%). The number of laparoscopic uterine and adnexal surgeries has increased annually (Figure 9). Laparoscopic procedure‐related complications occurred in 3340 patients (3.5%) in 2022 and 3952 patients (3.8%) in 2023. Furthermore, 215 patients (102 patients in 2022 and 113 patients in 2023) required conversion to open surgery.

### 12.2 Hysteroscopic Surgery

Hysteroscopic surgery was performed on 216 054 patients between 1990 and 2023. The most common indication for hysteroscopy was endometrial polyps (115 707 patients, 53.6%), followed by submucosal myomas (80 155 patients, 37.1%). Complications occurred in 171 patients (1.0%) in 2022 and 191 patients (1.0%) in 2023.

### 12.3 Salpingoscopy

A total of 10 277 salpingoscopies were performed between 1990 and 2023. The most common indication for salpingoscopy was oviduct obstruction (6870 cases, 66.8%), followed by ovarian duct stenosis (2220 cases, 21.6%) and functional sterility (859 cases, 8.4%). Complications occurred in one case in 2022; however, no intraoperative or postoperative complications have been reported in 2023.

## 13 Urology

### 13.1 Selection of the Laparoscopic Urological Surgery Method

All 227 institutions employed the pneumoperitoneum method.

### 13.2 Single‐Port Laparoscopic Surgery

By the end of 2023, 5381 patients had undergone single‐port laparoscopic surgery. The most performed single‐port laparoscopic surgery was prostatectomy (1127 cases, 20.9%), followed by radical nephrectomy (927 cases, 17.2%) and kidney harvesting for transplantation (876 cases, 16.3%).

### 13.3 Port‐Site Recurrence

Port‐site recurrence was reported in four patients in 2022 and 2023 (renal cancer in two, bladder cancer in one, and prostatic cancer in one). One patient with port site recurrence underwent surgery. Two patients received systemic chemotherapy, and one patient received radiotherapy.

### 13.4 Laparoscopic Adrenal Gland Surgery

By the end of 2023, 23 790 patients had undergone laparoscopic adrenal gland surgery. Of these, 242 patients underwent robot‐assisted surgeries. The most common indication for laparoscopic adrenal gland surgery was primary aldosteronism (8174 cases, 34.4%), followed by pheochromocytoma (3923 cases, 16.5%) and Cushing's syndrome (3171 cases, 13.3%). Of these 23 790 patients, 660 (2.8%) experienced intraoperative complications and 318 (1.3%) required conversion to open surgery. Postoperative complications were reported in 352 patients (1.5%).

### 13.5 Laparoscopic Kidney and Urinary Tract Surgery

**FIGURE 11:**
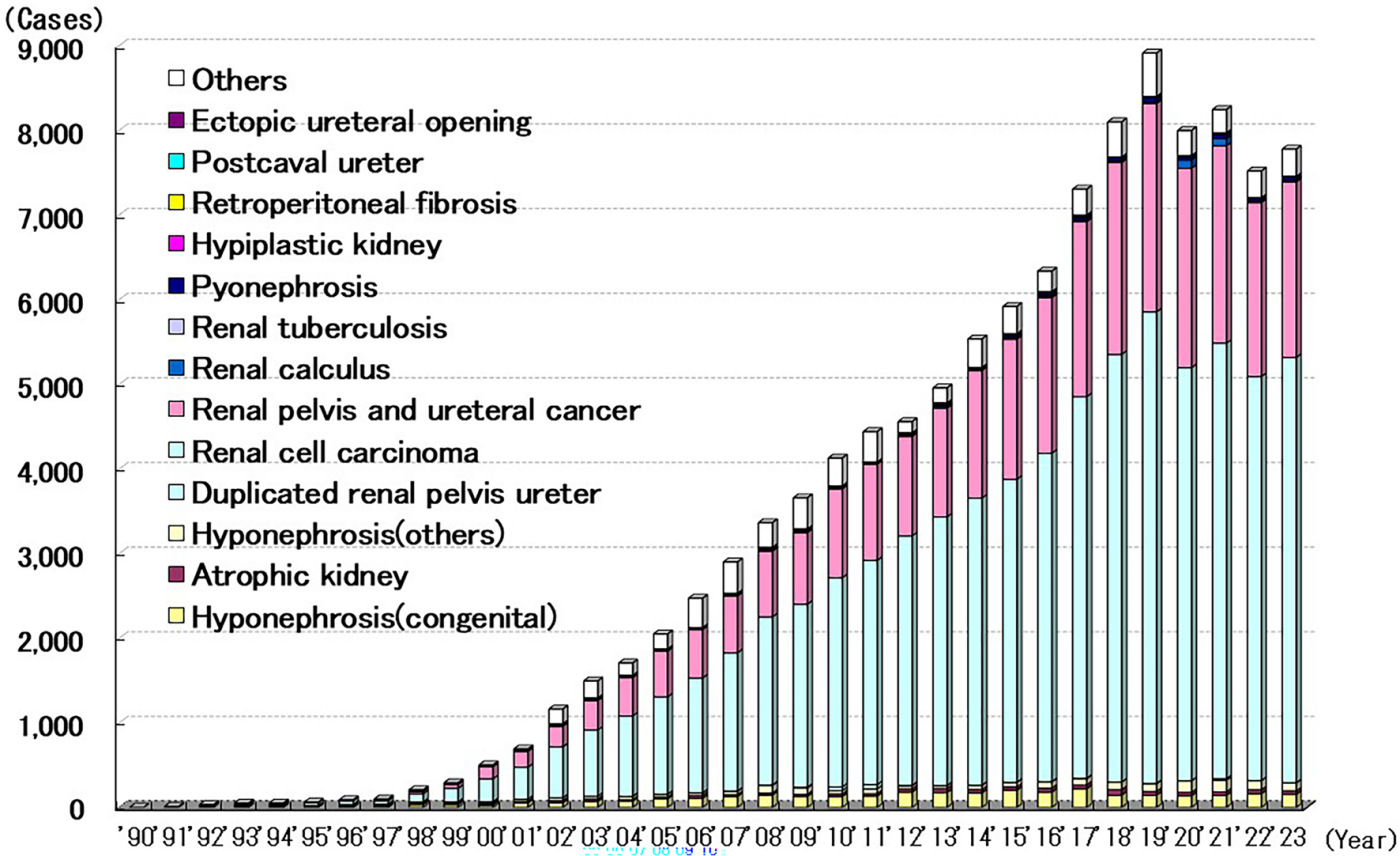
Annual number of laparoscopic procedures for kidney and urinary tract.

From 1990 to 2023, 112 610 laparoscopic renal and upper urinary tract surgeries were performed (Figure 11). The most common indication for laparoscopic renal and upper urinary tract surgery was renal cell carcinoma (68 714 cases, 61.0%), followed by renal pelvic and ureteral cancer (30 114 cases, 26.7%). The most performed procedure was laparoscopic radical nephrectomy (42 727/126139 cases; 33.9%). Robot‐assisted laparoscopic surgery was performed on 22 322 patients (17.7%). Intraoperative complications occurred in 2656 patients (2.1%), of whom 1463 required conversion to open surgery. Postoperative complications occurred in 2117 patients (1.7%).

### 13.6 Laparoscopic Surgery for Intra‐Abdominal Testis

A total of 2128 laparoscopic surgeries for intra‐abdominal testes were performed between 1990 and 2023. The most common indication was anarchism (760 cases, 35.7%), followed by laparoscopic orchiopexy (636 cases, 29.9%) and laparoscopic orchidectomy (475 cases, 22.3%).

### 13.7 Laparoscopic Varicocolectomy

Between 1990 and 2023, 2420 laparoscopic varicocelectomies were performed. Laparoscopic ligation of testicular veins with preservation of the testicular artery was performed in 1276 patients (52.7%).

### 13.8 Laparoscopic Surgery for Prostatic Cancer

Between 1990 and 2023, 107 512 laparoscopic surgeries for prostate cancer were performed. Robot‐assisted laparoscopic surgery was performed in 79 216 patients (73.7%). Intraoperative complications occurred in 1557 patients (1.4%), and 216 patients (0.2%) required conversion to open surgery. Postoperative complications occurred in 2017 patients (1.9%).

### 13.9 Laparoscopic Surgery for Other Urological Diseases

Between 1990 and 2023, 19 701 laparoscopic surgeries were performed. The most performed surgery was laparoscopic total cystectomy (7936 patients). Intra‐ and postoperative complications were observed in 359 patients in 2022 and 2023. In addition, 28 patients required conversion to open surgery.

## 14 Orthopedics

Between 2008 and 2023, 23 967 spinal endoscopic surgeries were performed. Most of these were posterior lumbar spinal surgeries, for which 21 653 spinal endoscopic surgeries (90.3%) were performed. The second‐most‐performed procedure was posterior cervical spinal surgery (1277 cases, 5.3%). The most common indication for posterior spinal surgery was a herniated disc (57.5%). Intraoperative complications occurred in 655 patients (2.7%), whereas postoperative complications occurred in 195 (0.8%). The mortality rate was 0%. One patient required conversion to open thoracotomy, and one patient required open laparotomy.

## 15 Plastic Surgery

A total of 7028 endoscopic surgeries were performed between 1994 and 2023. The most performed surgery was endoscopic carpal tunnel release (2407 patients, 38.0%), followed by endoscopic sinus surgery (1287 patients, 22.6%). Intraoperative complications occurred in 11 patients, and postoperative complications in 400 patients in 2022 and 2023.

## 16 Conclusion

The 17th JSES Nationwide Survey demonstrated that endoscopic surgery is widely performed and safely conducted across multiple surgical specialties in Japan. Despite the impact of the coronavirus disease (COVID‐19) pandemic in previous years, the number of endoscopic procedures performed has steadily recovered and increased in most clinical fields [9, 10]. Robot‐assisted surgery has shown remarkable growth across disciplines, reflecting ongoing technological advancements and broader clinical adoption. These nationwide multidisciplinary data provide an essential foundation for evaluating current practice patterns and will contribute to the further development, standardization, and safe dissemination of endoscopic surgery in Japan.

## Funding

The authors have nothing to report.

## Conflicts of Interest

The authors declare no conflicts of interest.

## Data Availability

The data of this article is based on the results of the 16th National Survey conducted in 2022–2023 by the JSES.
